# Bone Marrow Derived Mesenchymal Stem Cells Incorporate into the Prostate during Regrowth

**DOI:** 10.1371/journal.pone.0012920

**Published:** 2010-09-23

**Authors:** Veronica R. Placencio, Xiaohong Li, Taylor P. Sherrill, Gloria Fritz, Neil A. Bhowmick

**Affiliations:** 1 Department of Cancer Biology, Vanderbilt University, Nashville, Tennessee, United States of America; 2 Department of Urologic Surgery, Vanderbilt University, Nashville, Tennessee, United States of America; 3 Department of Pulmonary and Critical Care Medicine, Vanderbilt University, Nashville, Tennessee, United States of America; 4 Samuel Oschin Comprehensive Cancer Institute, Cedars-Sinai Medical Center, Los Angeles, California, United States of America; East Carolina University, United States of America

## Abstract

**Background:**

Prostate cancer recurrence involves increased growth of cancer epithelial cells, as androgen dependent prostate cancer progresses to castrate resistant prostate cancer (CRPC) following initial therapy. Understanding CRPC prostate regrowth will provide opportunities for new cancer therapies to treat advanced disease.

**Methodology/Principal Findings:**

Elevated chemokine expression in the prostate stroma of a castrate resistant mouse model, Tgfbr2^fspKO^, prompted us to look at the involvement of bone marrow derived cells (BMDCs) in prostate regrowth. We identified bone marrow cells recruited to the prostate in GFP-chimeric mice. A dramatic increase in BMDC recruitment for prostate regrowth occurred three days after exogenous testosterone implantation. Recruitment led to incorporation of BMDCs within the prostate epithelia. Immunofluorescence staining suggested BMDCs in the prostate coexpressed androgen receptor; p63, a basal epithelial marker; and cytokeratin 8, a luminal epithelial marker. A subset of the BMDC population, mesenchymal stem cells (MSCs), were specifically found to be incorporated in the prostate at its greatest time of remodeling. Rosa26 expressing MSCs injected into GFP mice supported MSC fusion with resident prostate epithelial cells through co-localization of β-galactosidase and GFP during regrowth. In a human C4-2B xenograft model of CRPC, MSCs were specifically recruited. Injection of GFP-labeled MSCs supported C4-2B tumor progression by potentiating canonical Wnt signaling. The use of MSCs as a targeted delivery vector for the exogenously expressed Wnt antagonist, secreted frizzled related protein-2 (SFRP2), reduced tumor growth, increased apoptosis and potentiated tumor necrosis.

**Conclusions/Significance:**

Mesenchymal stem cells fuse with prostate epithelia during the process of prostate regrowth. MSCs recruited to the regrowing prostate can be used as a vehicle for transporting genetic information with potential therapeutic effects on castrate resistant prostate cancer, for instance by antagonizing Wnt signaling through SFRP2.

## Introduction

Prostate cancer mortality continues to rise as the aging population expands. After surgical intervention, radiation, and androgen ablation therapy, cancer recurrence is androgen-independent and is termed castrate resistant prostate cancer (CRPC). Patients with CRPC are primarily given palliative care since conventional chemotherapeutics do little to delay mortality from the disease. Prostate growth and regrowth has been the subject of stem cell studies, but not with respect to recruited cell types [1], [2]. We used transgenic mouse models to help understand the involvement of recruited cells in prostate regrowth.

Prostatic epithelial Wnt signaling was identified in transgenic mouse models of CRPC. One such model we developed had a conditional stromal knockout of the TGF-β type II receptor, termed Tgfbr2^fspKO^, [3]. FSP1-Cre enabled targeted recombination in mesenchymal-derived cells, including fibroblasts [3], [4]. The prostatic stroma of Tgfbr2^fspKO^ mice independently contributed to transformation of the adjacent epithelia as well as castrate resistance. The mechanism for the castrate resistant phenotype was associated with stromal fibroblastic expression of Wnt ligands, that in turn activated canonical Wnt signaling in the epithelia [5], [6]. Adenoviral transduction of the Wnt signaling antagonist, secreted frizzled related protein-2 (SFRP2) restored castrate responsiveness in the Tgfbr2^fspKO^ mice [5]. However, systemic SFRP2-adenovirus treatment in Tgfbr2^fspKO^ mice was associated with morbidity. Another model with prostatic epithelial expression of constitutively activated β-catenin developed HGPIN and sustained growth following castration [7]. Additionally, human CRPC C4-2B cells reportedly have elevated autocrine Wnt signaling compared to their androgen dependent parent cell line, LNCaP [8].

CRPC, tissue remodeling, and cancer progression are generally associated with the recruitment of bone marrow derived cells [9], [10], [11]. In a mouse model of damaged liver, during regeneration the recruited BMDCs contributed to the new liver epithelia [12]. Similar BMDC incorporation occurs in gastrointestinal epithelia associated with elevated proliferation and inflammation [13], [14]. A specialized type of BMDCs, mesenchymal stem cells (MSCs), are recruited to sites of inflammation and proliferation including wound healing and cancer [15]. Multipotent MSCs can differentiate into multiple cell types including osteoblasts, chondrocytes, adipocytes, and fibroblasts [16]. In various models of cancer, MSCs potentiate disease progression. In a model of breast cancer, MSCs promoted metastasis [17]. In a model of prostate cancer metastasis, manipulating MSCs to deliver the urokinase-type plasminogen antagonist amino-terminal fragment by co-injection with PC3 cells in the bone decreased tumor angiogenesis and osteolytic activity [18]. MSCs home to tumors as a result of cytokine and chemokine expression in the tumor microenvironment. The efficacy of MSCs as a therapeutic tool has been tested through the targeted delivery of exogenously expressed soluble factors [19], [20]. For example, patients with heart damage have received MSCs that home to damaged and necrotic tissue for wound repair [21]. We rationalized that MSC therapy may benefit patients with CRPC in a similar manner.

We examined BMDC recruitment to the prostate during regrowth. Co-expression of prostate markers with BMDCs suggested that these recruited cells were also incorporated into the prostate epithelia. We further identified MSCs fusing with prostatic epithelia. Using an orthotopic C4-2B xenograft model system, we found that recruited MSCs could further contribute to tumor progression by enhanced Wnt signaling. The overexpression of SFRP2 by MSCs homed to the tumors and restored tumor responsiveness to castration.

## Results

Previous studies with the Tgfbr2^fspKO^ mouse model showed that the conditional stromal knockout of TGF-β signaling led to CRPC [5]. To fully understand the stromal changes that led to the development of CRPC, we isolated prostate stromal RNA through laser capture microdissection for microarray analysis and compared Tgfbr2^fspKO^ to control Tgfbr2^FloxE2/FloxE2^ mouse stroma. The microarray data has been deposited in the NCBI Gene Expression Omnibus (GEO, GSE22130). This revealed the differential regulation of numerous cytokines and chemokines in Tgfbr2^fspKO^ compared to control Tgfbr2^FloxE2/FloxE2^ mouse stroma (Table 1). Microenvironments rich in chemokine and cytokine signaling recruit many cell types, including BMDCs [22].

**Table 1:** Selected List of Chemokines.

Primary Sequence Name	Average Fold Change
Ccl12	−3.5079
Ccl19	2.9717
Ccl5	−70.1794
Ccl6	−26.8950
Ccl8	−39.9607
Ccl9	−11.6369
Csf1r	−9.6176
Csf2	162.4770
Cx3cl1	−25.0406
Cxcl10	−25.4580
Cxcl12	77.3219
Cxcl13	−47.1429
Cxcl16	−20.5419
Cxcl9	−49.5591
Epo	40.0280
Ifna12	38.8468
Ifnz	159.8488
Il18	−2.1320
Il18bp	−13.0765
Il1f8	3.3513
Il28	10.2366
Il4	−3.7385
Il6st	−31.2994

Selected chemokines altered in Tgfbr2^fspKO^ mouse stroma compared to Tgfbr2^FloxE2/FloxE2^ mouse stroma. Negative values indicate the chemokine was downregulated in the Tgfbr2^FloxE2/FloxE2^ stroma and upregulated in the Tgfbr2^fspKO^ stroma. Positive values indicated the chemokine was upregulated in the Tgfbr2^FloxE2/FloxE2^ stroma and downregulated in the Tgfbr2^fspKO^ stroma. Values are averaged from three independent data sets. Each value had >2-fold difference and a p-value <0.01.

**Figure 1:**
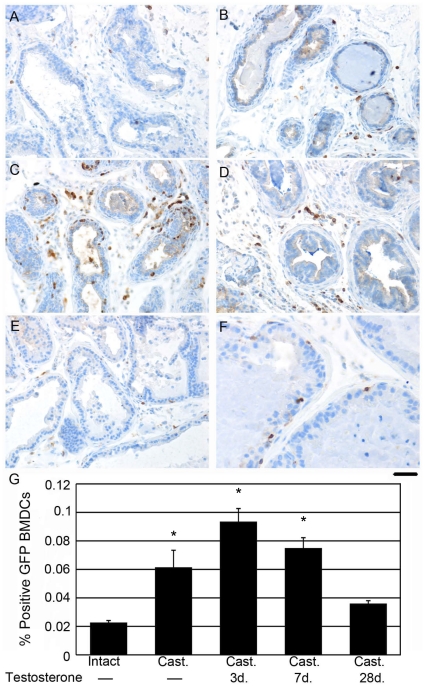
BMDCs were recruited to the prostate during regrowth. GFP-BMDC (brown) recruitment was localized in prostates of GFP chimeric mice. BMDCs were quantitated in prostates of A) intact mice, B) two months post-castration, C) castrated mice supplemented with exogenous testosterone for three days, D) castrated mice supplemented with exogenous testosterone for seven days, and E) castrated mice supplemented with exogenous testosterone for 28 days. F) Higher magnification of castrated prostates supplemented with exogenous testosterone for 28 days indicated BMDC recruitment to the epithelial ductal compartment. Scale bar indicates 50 µm for panels A–E and 25 µm for panel F. G) The bar graph quantifies the percentage of GFP-positive BMDCs recruited to the prostate at the indicated time points during regrowth. Oneway ANOVA was performed to test differences between the experimental and control groups (n = 3 per group), followed by Dunnett's post-hoc tests. Statistically significant values are indicated with * in the bar graph. Error bars indicate standard deviation. The omnibus of F statistic from the ANOVA was 43.3 with 4 and 10 degrees of freedom (p<0.0001). Dunnett's post-hoc tests showed differences between intact (mean  = 0.0227) and castrated (cast) (mean  = 0.0615) (p<0.0003), cast +3dT (mean  = 0.00936) (p<0.0001), and cast +7dT (mean  = 0.0750) (P<0.0001). The intact group did not differ from cast +28dT (mean  = 0.0360) (p<0.1629).

BMDCs are recruited to the prostate during cancer development. Since BMDCs are also recruited during tissue remodeling, we wanted to identify their role in prostate regrowth following castration. Previous studies have described the histologic identification of macrophage and monocytes recruited in prostate cancer [11], [23]. To better localize BMDCs during prostate regrowth we generated GFP-bone marrow chimeric wild type mice, which were either intact or castrated. Prostate regrowth was monitored at day zero, three, seven, and 28 days following implantation of exogenous testosterone. Immunohistochemical GFP localization identified BMDCs recruited to the prostate during regrowth in response to castration (Figure 1). Intact prostates had low basal BMDC recruitment (Figure 1A). Castration induced a slight, but statistically significant increase in recruitment (Figure 1B). Three and seven days following testosterone supplementation, prostates reached the highest levels of recruitment (Figure 1C, D). At 28 days following testosterone supplementation, when the prostate was fully regrown, the level of detectible BMDCs had fallen, comparable to basal levels (Figure 1E–F). Of particular interest was the appearance of BMDCs residing within the epithelial compartment (Figure 1F). BMDC recruitment during active prostate remodeling is quantitated in Figure 1G. BMDC incorporation in the epithelial compartment was observed throughout the duration of recruitment from castration to full regrowth. However, the presence of BMDCs at four weeks when the prostates had resumed normal homeostasis suggested that BMDCs directly contributed to the regenerated prostate tissue.

**Figure 2:**
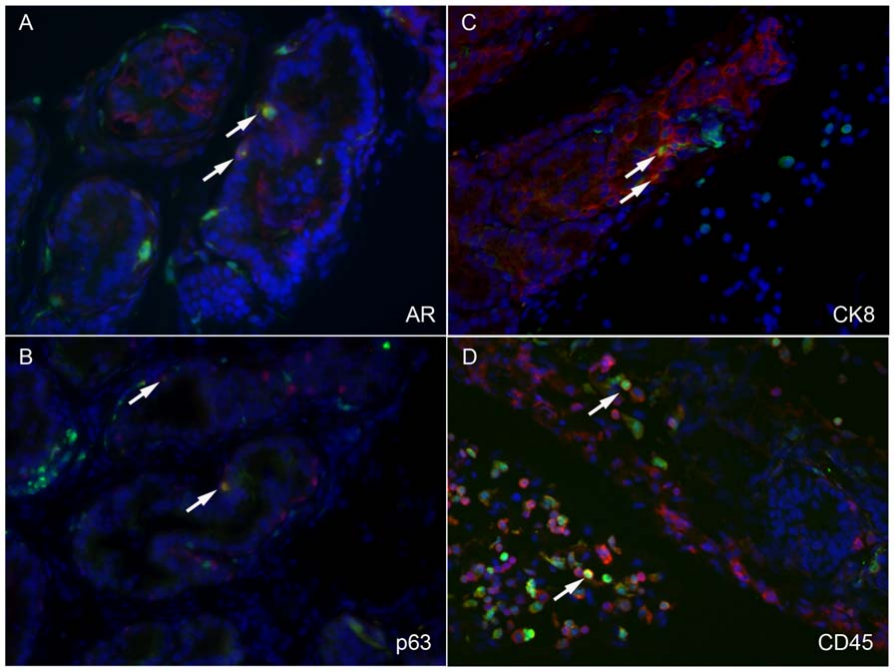
BMDCs incorporated into the prostate during regrowth. Immunofluorescence staining localized GFP-BMDCs (green) with prostate-specific markers (red). DAPI stained the nuclei (blue). A) Androgen receptor, B) p63, and C) cytokeratin 8 were expressed in recruited GFP-BMDCs. D) GFP-BMDCs had minimal co-expression of the hematopoietic marker, CD45.

We characterized the BMDCs that appeared to incorporate into the prostate epithelia. GFP expression was immunolocalized with prostate tissue markers (Figure 2). We found that co-expression of GFP with androgen receptor indicated the prostate cell lineage of stromal or epithelial cells (Figure 2A). Furthermore, basal and prostate luminal epithelial cells identified by p63 and cytokeratin 8, respectively, also co-expressed GFP (Figure 2B, C). We also noted limited co-expression of GFP with CD45, indicating hematopoietic lineage cells (Figure 2D). These results indicated that although a large population of BMDCs is recruited to the prostate, comparatively smaller populations of the total BMDC population seem to incorporate into the tissue long term.

**Figure 3:**
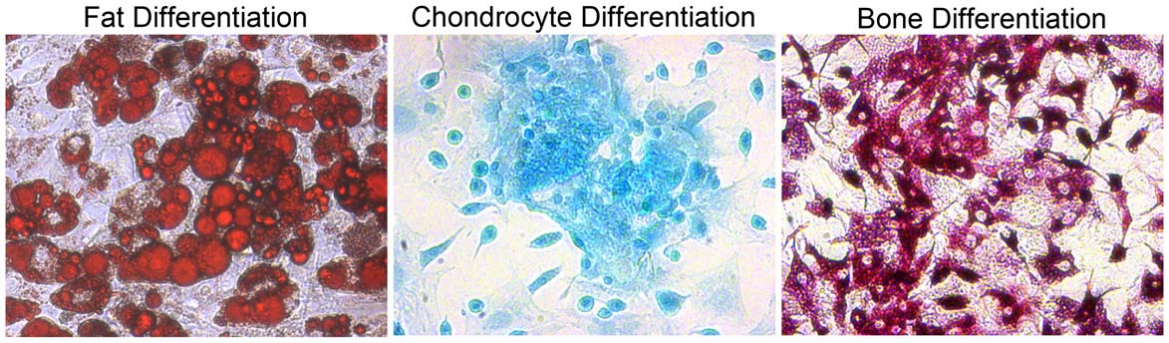
MSCs were functionally verified. Functional assays verified tri-lineage differentiation potential of mesenchymal stem cells after culturing for three weeks. Adipogenic differentiation was verified by staining with Oil Red-O; lipid droplets stained a red color. Chondrogenic differentiation was verified by staining with alcian blue; matrix sulfated proteoglycans stained a turquoise blue color. Osteogenic differentiation was verified by alkaline phosphatase staining; osteogenic cells stained a deep fuschia color.

We hypothesized that at least a subset of the incorporated BMDCs were mesenchymal stem cells (MSCs) based on evidence that 1) MSCs are less than one percent of the total bone marrow population of cells, 2) MSCs are reported to home to sites of active tissue remodeling, and 3) MSCs are known to incorporate into regenerated tissues [12], [24]. We next determined the recruitment of MSCs to the prostate during regrowth. We generated primary cultured MSCs from adult chicken β-actin-GFP mouse bone marrow [25]. The MSC trilineage differentiation potential was verified. The MSCs were shown to differentiate into adipocytes, as indicated with oil red-O staining, chondrocytes indicated with alcian blue staining, and osteocytes indicated by alkaline phosphatase staining (Figure 3). The multipotential MSCs were tested for the expression of known chemokine receptors. As expected, the MSCs expressed CCR5, CCR2, and CXCR4. Next, we measured the expression of corresponding chemokines in prostates during regrowth, to determine a mechanism for MSC homing. We isolated RNA from castrated prostates and castrated prostates given testosterone for three days. CCL5 was confirmed to be elevated 2.6-fold during prostate regrowth (Student's *t*-test, p = 0.0224) compared to castrated controls. However, CCL2 and SDF-1 were not significantly elevated during regrowth. Thus, the CCR5-CCL5 chemokine signaling axis is a potential mechanism for MSC recruitment during prostate regrowth.

**Figure 4:**
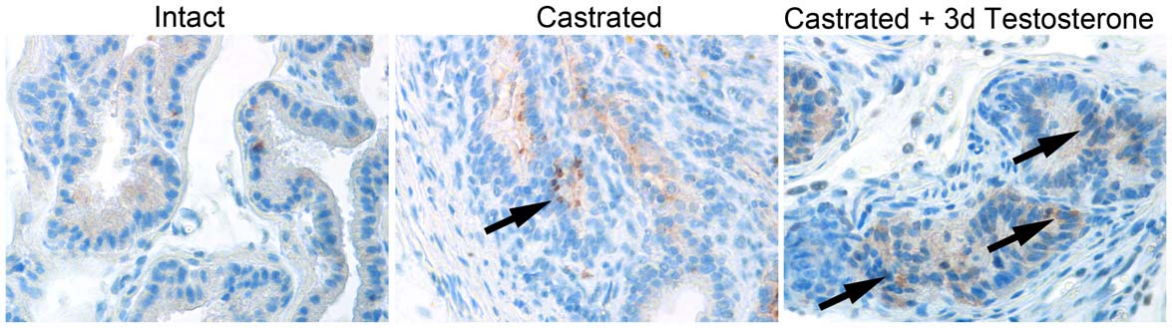
MSCs were recruited to the prostate during regrowth. GFP-MSCs (brown) were recruited to the prostates of mice at the indicated time points during regrowth. MSC recruitment was focal, and quantitation was performed for selected fields of view with detectable MSCs. Oneway Welch ANOVA allowing for unequal variances was used to test differences between the experimental groups and the intact group (n = 20), followed by a two-sample t tests with unequal variances. The omnibus of statistic from the ANOVA was 89.0 (p<0.0001). Two-sample t tests with unequal variances tests showed a statistically significant increase between intact (mean  = 7.25 cells per field of view) and castrated (mean  = 25.75 cells, probability <0.0001) and castrated +3dT (mean  = 38.55 cells, probability <0.0001).

GFP-tagged MSCs were injected into mice to identify MSC recruitment to the prostate. We used intact or castrated host mice that were given exogenous testosterone for zero or three days, correlating with the highest level of BMDC recruitment. We then monitored MSC recruitment by GFP immunohistochemical staining and found a pattern similar to that seen with BMDC recruitment (Figure 4). The GFP-MSCs were recruited to intact prostates at low levels (mean = 7.25 cells per field of view). Castrated mice had elevated MSC recruitment to the epithelial compartment of the prostate (mean = 25.75). Further elevation in MSC recruitment was observed following three days of testosterone supplementation (mean = 38.55). Importantly, examination of other tissues of the same mice (liver, intestine) revealed no detectible GFP-MSCs. Thus, the bone marrow derived MSC population could selectively incorporate into the prostate during regrowth. The fusion or transdifferentiation of MSCs is reported in other model systems [26], [27].

**Figure 5:**
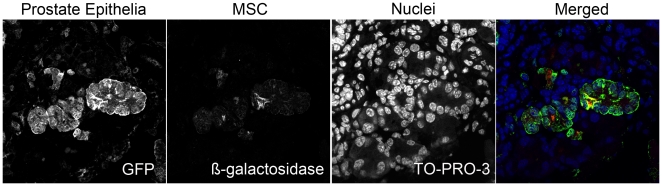
MSCs fused to the prostate during regrowth. β-galactosidase-expressing MSCs were injected into castrated GFP mice supplemented with exogenous testosterone for three days. Confocal microscopy showed colocalization of β-galactosidase-expressing MSCs (red) with GFP prostate ducts (green). Nuclei were stained with TO-PRO-3 (blue). Merged color panel illustrates colocalization of MSCs (red) with prostate epithelia (green), indicated by yellow. Fusion occurred in 48.9% of recruited MSCs.

We tested the potential of using MSC cell fusion or differentiation as a mechanism for targeting genetic material to CRPC. We used chicken β-actin-GFP mice as hosts for MSCs obtained from FSP1-Cre/Rosa26 mice. The development of FSP1-Cre/Rosa26 mice enabled MSC lineage tracing through the expression of β-galactosidase. Prostates of castrated mice were visualized by confocal fluorescent microscopy following three days of testosterone supplementation. We identified prostate epithelial cells that co-expressed GFP with β-galactosidase as an indication of fusion of the MSCs to the prostate epithelia (Figure 5). We calculated the percentage of recruited MSCs shown to undergo fusion at 48.9% (n = 5 mice, 20 fields of view).

**Figure 6:**
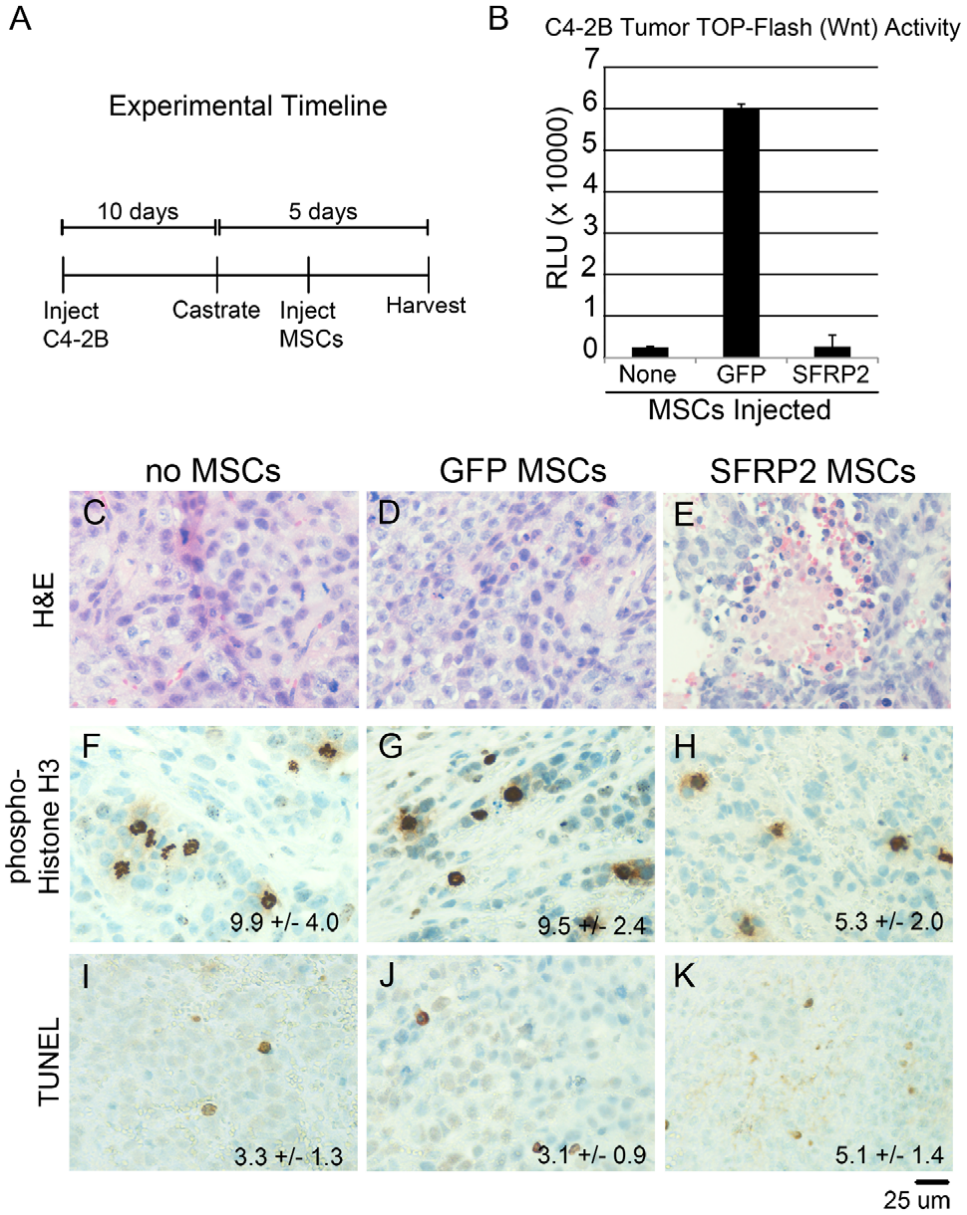
GFP-MSCs recruited to human CRPC xenografts enhanced Wnt signaling. A) The experimental setup is summarized in the timeline. C4-2B cells were orthotopically injected into the prostates of SCID mice. After 10 days the mice were castrated and MSCs were given 24 hours following castration. The tumors were harvested five days following castration. B) TOP-Flash luciferase activity was measured in C4-2B tumors. GFP-MSCs increased Wnt activity within the tumors by 24-fold compared to control uninjected tumors. SFRP2-MSCs suppressed Wnt activity comparable to basal levels. C4-2B tumors were sectioned and stained for histological analysis five days following castration. C4-2B tumors had necrosis in the C) absence of MSCs, D) GFP-MSCs, and E) the greatest in SFRP2-MSC injected mice. F) Basal levels of phosphorylated histone-H3 were observed in tumors without MSCs and G) GFP-MSCs. H) Proliferation was significantly decreased in tumors with SFRP2-MSCs (p value  = 0.0069). I) Basal levels of TUNEL-positive apoptosis were found in tumors without MSCs and J) GFP-MSCs. K) A significant increase in apoptosis was seen in tumors with SFRP2-MSCs (p value  = 0.0066). Areas of necrosis were avoided in quantitating TUNEL positive cells.

To further explore the role of MSCs in CRPC growth and their potential for targeted therapy, we used the C4-2B human CRPC cell line, well characterized to grow as xenografts in castrated host mice [28]. Based on the documented importance of Wnt signaling in CRPC [29], [30], we stably transduced C4-2B cells with a TCF/LEF responsive luciferase reporter lentivirus. This allowed us to monitor canonical Wnt activity within the tumor epithelia. We then transduced MSCs with either GFP adenovirus as a control or SFRP2 adenovirus. We hypothesized that MSCs could home to C4-2B tumors and secrete SFRP2 to inhibit tumor progression. To generate the CRPC model, we orthotopically grafted C4-2B cells into each of the anterior lobes of SCID mouse prostates. The experimental timeline is summarized (Figure 6A). After ten days of tumor growth, the host mice were castrated. Serum testing indicated continued expression of PSA in C4-2B tumor bearing mice following castration, as expected. To test the efficacy of MSCs as a therapeutic tool for CRPC, we subsequently injected host mice with vehicle, GFP-transduced MSCs, or SFRP2-transduced MSCs and harvested the tumors five days after castration.

After harvesting the C4-2B tumors, we measured TCF/LEF-luciferase activity, proliferation, and apoptosis. Analysis of luciferase expression from the lysed tumors revealed that mice given GFP-MSCs showed a 24-fold increase of canonical Wnt activity within the C4-2B tumor epithelia compared to controls (Figure 6B). Tumors from mice injected with SFRP2-MSCs had no difference in canonical Wnt activity compared to controls. This surprising finding suggested that five days following castration, wild type MSCs contribute to CRPC by enhancing Wnt signaling activity. Manipulating the MSCs to secrete SFRP2 caused reduced proliferation and elevated apoptosis associated with increased necrosis within the tumors.

H&E staining indicated focal areas of necrosis in control C4-2B tumors and in mice given GFP-MSCs (Figure 6C, D). The C4-2B tumors in mice given SFRP2-MSCs had comparatively greater areas of necrosis (Figure 6E). Tumor proliferation was analyzed by immunohistochemical localization of phosphorylated histone-H3, indicating cells undergoing mitotic division (Figure 6F-H). The mitotic rates of control and GFP-MSC-associated tumors were similar (Figure 6D, E). However, the mitotic rate in SFRP2-MSC-associated tumors was significantly lower than in control tumors (p value  = 0.0069, Figure 6F). We next analyzed apoptosis in these tumors using TUNEL staining (Figure 6G-I). Control and GFP-MSC-associated tumors had similar apoptotic cell numbers (Figure 6G, H). However, SFRP2-MSC tumors had a significant increase in apoptotic cells (p value  = 0.0066, Figure 6I). Together, we found MSC recruitment to CRPC xenografts to support Wnt signaling and the targeted antagonism of Wnt signaling by MSCs can potentiate reduced tumor growth.

## Discussion

**Figure 7:**
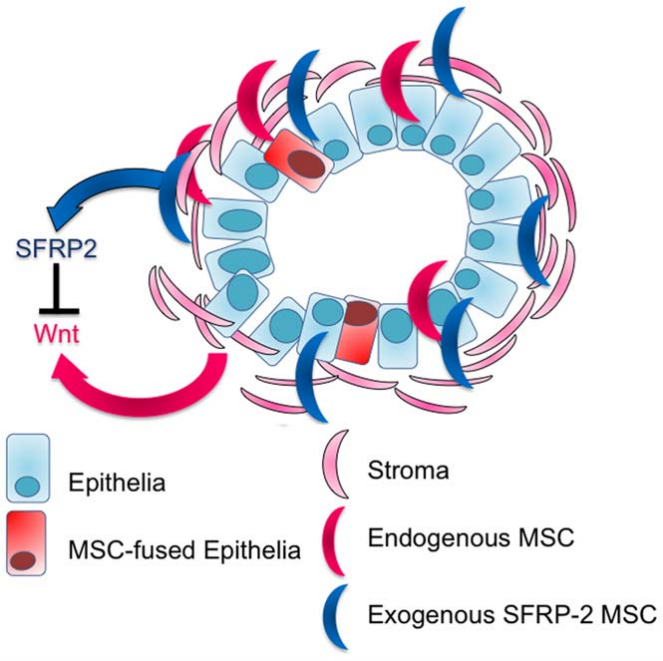
MSCs contribute to CRPC. This diagram represents a transformed prostate gland. Rounded turquoise rectangles indicate epithelial cells with an irregular nuclear shape and the larger pink half moon shapes represent the stromal compartment. The stroma surrounds the ducts, and is invaded by the transformed epithelia. The recruited endogenous-MSCs (red) contribute to Wnt signaling in CRPC. Whereas, the recruited therapeutic-MSCs that express SFRP-2 (blue) antagonize Wnt signaling in CRPC. Rounded red rectangles indicate epithelial cells that have fused with recruited MSCs.

Recruited BMDCs enhanced tumor progression through paracrine Wnt signaling. We found that the highest levels of BMDC recruitment occurred during prostate regrowth at three days. Interestingly, previous reports indicated that the highest level of prostate proliferation also occurs three days following testosterone re-introduction, suggesting that peak tissue remodeling corresponds to greatest recruitment of BMDCs. This was to be expected, since BMDCs include inflammatory responsive cells such as leukocytes and macrophages, associated with clearing dead cells. However, this study demonstrated that a sub-population of BMDCs, MSCs, are recruited to potentiate re-growth. MSCs may provide growth factors that aid cancer progression in this complex signaling microenvironment (Figure 7). We were able to employ MSCs to target regrowing prostate tissue and deliver SFRP2, antagonizing Wnt-mediated tumor progression.

Based on the microarray analysis of the Tgfbr2^fspKO^ CRPC mouse model, (Table 1) and xenografted human CRPC C4-2B cells (Figure 6) it is likely that MSCs were recruited during regrowth through the CCL5-CCR5 axis. To broaden our understanding of mechanisms of CRPC, signaling pathway alterations based on LCM-microarray from Tgfbr2^fspKO^ compared to Tgfbr2^floxE2/floxE2^ mouse stroma were analyzed. Interestingly, many more chemokines were upregulated than downregulated in the Tgfbr2^fspKO^ prostate stroma. Of particular interest, CCL5 had a 70-fold increase in Tgfbr2^fspKO^ stroma (Table 1). CCL5 is known to be upregulated in prostate cancer [31]. It has also been shown to be a potent recruiter of BMDCs, including MSCs [32]. A significant alteration of chemokine signaling within the tumor microenvironment was likely to result in BMDC recruitment. The multiple co-morbidities of the Tgfbr2^fspKO^ mice [3], [5], [6], [33] prevented us from generating chimeric mice using these as the host to track BMDC recruitment to the prostate. Therefore, we looked at recruitment during prostate regrowth in wild type mice as a way to understand this aspect of CRPC.

We demonstrated how the recruited mesenchymal stem cells home to and contribute to CRPC by enhancing Wnt signaling within the tumor epithelia. Using MSCs as a delivery tool for secreted frizzled related protein 2 suppressed the enhanced Wnt signaling in the tumor epithelia to effectively increase apoptosis. MSCs may be used to deliver other gene therapeutics to enable CRPC regression. Using MSCs as a targeted delivery tool is also a way to avoid the toxicity associated with other less specific therapies. When targeting signaling pathways that have systemic effects, such as Wnt, it is especially necessary to avoid widespread repercussions.

We showed that MSCs incorporate themselves into the prostate epithelia through fusion. It is likely that the total number of recruited MSCs is over-estimated due to the experimental conditions of administering one million MSCs. However, the percentage of recruited MSCs observed to undergo fusion (48.9%), is likely similar to endogenous fusion events, respectively. Other studies have demonstrated how hematopoietic stem cells incorporate into the gastrointestinal epithelia through fusion during inflammation and proliferation [34]. Prostate injury from vaccinia virus infection can cause inflammation and glandular disruption, resulting in BMDC reconstitution of 4% of the prostate epithelium [35]. Other studies have shown BMDCs recruited to and then incorporate into resulting epithelial tissues found in lung, liver, skin, heart, and the gastrointestinal tract [36], [37], [38]. Cancer recurrence is linked to cell fusion [39]. CRPC cells have properties including increased drug resistance, increased resistance to apoptosis, and enhanced malignancy that are known to result from cells that have undergone cell fusion [39]. We did not rule out the possibility of transdifferentiation into prostate epithelial lineages. The chicken β-actin-GFP mice used in the fusion experiment had focal expression of GFP in the prostate. Therefore, the presence of β-galactosidase expressing MSCs in the absence of GFP could indicate transdifferentiation of the MSCs that incorporated into the prostate epithelia or fusion that we could not visualize due to the lack of GFP expression in those prostate epithelial cells. It is possible that both fusion and transdifferentiation are mechanisms of MSC incorporation into the prostate epithelia. Others have suggested that cancer stem cells may arise from the fusion of BMDCs with cancer cells, giving rise to cells more capable of evading immune control. While our study could not look at every possibility, we demonstrated the involvement of MSCs in the cancer microenvironment and the possibility of using MSCs to deliver therapeutic genes to control prostate cancer.

In the C4-2B xenograft studies, the recruitment of GFP-MSCs enhanced Wnt activity within the tumors. However, there was little effect on proliferation, apoptosis, or tumor size in C4-2B xenografts in the presence or absence of GFP-MSC recruitment. This may be because C4-2B cells express high levels of Wnt ligands endogenously, masking the effects of Wnt expression by the GFP-MSCs. This led us to hypothesize that MSCs may have a greater influence on androgen dependent tumors with lower amounts of Wnt that would be more sensitive to additional Wnt produced in the prostate microenvironment. By understanding how these MSC act upon cancer cells and contribute to the tumor microenvironment, we could learn to manipulate these cells to counteract their cancer promoting effects. We had some success in suppressing C4-2B tumor proliferation and promoting apoptosis with MSC-targeted SFRP2 expression. However, we did not observe a significant reduction in tumor size. Targeting Wnt signaling is just one route for suppressing cancer progression. An approach targeting multiple signaling pathways is likely to be more effective in this late stage of cancer progression to provide a lasting effect on tumor size.

Collectively, these studies demonstrated that BMDCs are involved in prostate regrowth and cancer progression. BMDCs, and in particular MSCs, are recruited to tissues undergoing active remodeling, including the cancer microenvironment. Given the innate cancer-homing capabilities of MSCs, it may be possible to use these cells to treat CRPC with therapeutic gene delivery.

## Materials and Methods

### Ethics Statement

The Vanderbilt Institutional Animal Care and Use Committee approved all animal procedures (M/06/236).

### Mice and Fetal Liver Transplants

Harlan C57BL/6 wild type mice were purchased (Harlan, Indianapolis, IN). Chicken β-actin-GFP mice were purchased from Jackson Laboratories and GFP expression was confirmed by visualization with UV light. Tgfbr2^floxE2/floxE2^ [40] and Tgfbr2^fspKO^ [3] mice with C57BL/6 background were generated as previously described. Fsp-Cre+/Rosa26 mice enabled β-gal visualization of cells after Cre-mediated recombination. All mice were genotyped from ear punch biopsies as previously reported [40], [41], [42]. Fetal liver transplants (FLTs) were performed as previously described [43]. Briefly, E14 embryonic livers from GFP+ mice were lysed into single cell suspensions for tail vein injections (2×10^6^ cells per mouse) into lethally irradiated recipient male mice using a [^137^Cs] gamma source by giving a split dose of 1200 rads. The host mice were either intact or castrated four weeks prior to the FLT procedure. To minimize infection from a diminished immune system, mice were kept on acidified antibiotic water for two weeks before and four weeks after FLTs. Four weeks after FLTs, prostate regrowth was monitored following subcutaneous implantations of testosterone pellets.

### Laser Capture Microdissection and RNA Isolation for Microarray

Tgfbr2^floxE2/floxE2^ and Tgfbr2^fspKO^ mouse prostates were dissected and frozen in liquid nitrogen. Cryosections of the prostates were used for laser capture microdissection of the stroma. RNA was isolated using a µMACS mRNA isolation kit following the manufacturer's protocol (Miltenyi Biotec, Auburn, CA). Samples were lysed in SuperAmp Lysis Buffer and sent for processing and microarray analysis. Miltenyi Biotec amplified the RNA, produced cDNA, and hybridized to Agilent whole genome oligo microarrays. Fluorescent signals were detected and Agilent Feature Extraction Software was used to read and process the microarray image files. Gene lists were given as normalized Cy5/Cy3-fold changes. The microarray data sets were submitted to the NCBI GEO database accession number GSE22130. The selected table included fold change values averaged from three data sets per genotype, each having a fold change >2 and p-value <0.01.

### MSC Generation and Verification

Bone marrow derived mesenchymal stem cells were derived as previously described [25], [44], [45]. Bone marrow was flushed from mice femurs and tibias aged eight to twelve weeks. Red blood cells were lysed and the remaining bone marrow cells plated in MSC expansion media [25], [45]. Adherent MSCs were selected and expanded for ten days. Cells were then trypsinized and plated for differentiation assays or used for tail vein injections into host mice (10^6^ cells injected per mouse).

Osteogenic differentiation of MSCs was performed as previously described [46]. MSCs were switched to osteogenic inducing media for three weeks, consisting of high glucose DMEM supplemented with 10% fetal bovine serum, 100 µM dexamethasone, 0.1 mM ascorbic acid, and 10 nM β-glycerophosphate. Osteogenic differentiation was confirmed by alkaline phosphatase staining according to the manufacturer's directions (Sigma-Aldrich, St. Louis, MO).

Chondrogenic differentiation of MSCs was performed as previously described [46]. MSCs were switched to chondrogenic inducing media for three weeks, consisting of high glucose DMEM supplemented with 10% fetal bovine serum, 100 µM dexamethasone, and 0.1 µg/mL TGF-β. Chondrogenic differentiation was confirmed by alcian blue (pH 1.0) staining. Cells were fixed for 20 min at RT in 10% formalin, washed with PBS, stained with alcian blue for 20 min and washed with PBS.

Adipogenic differentiation of MSCs was performed as previously described [46]. MSCs were switched to adipogenic inducing media for three weeks, consisting of high glucose DMEM supplemented with 10% fetal bovine serum, 10 µM dexamethasone, 10 µg/mL insulin, and 100 µg/mL IBMX. Adipogenic differentiation was confirmed by Oil Red-O staining.

### Immunohistochemistry and Immunofluorescence

Tissues were fixed with 4% paraformaldehyde or 10% formalin, embedded in paraffin, and sectioned for histological analysis. GFP (1∶1000, Santa Cruz, CA) and phosphorylated-histone H3 (1∶500, Upstate, Temecula, CA) immunohistochemistry was performed using antigen retrieval with antigen unmasking solution (Vector Laboratories, Burlngame, CA) diluted 1∶100. Following primary antibody incubation overnight, Dako Cytomation Universal or Rabbit kits were used for the secondary antibody and development with DAB. TUNEL staining was performed using the ApopTag Peroxidase In Situ Apoptosis Detection Kit (Millipore, Temecula, CA) as directed. Immunohistochemical staining was quantitated by taking a ratio of positively stained cells per field (400×) MetaMorph 7.6 software was used to help quantitate the immunohistochemical staining. Statistical significance was determined by two tailed Student's *t* test.

Tissues were fixed with 4% paraformaldehyde or 10% formalin, embedded in paraffin, and sectioned for histological analysis. Antigen retrieval with antigen unmasking solution (Vector Laboratories, Burlngame, CA) diluted 1∶100 was performed. Staining was performed for GFP (1∶100, Santa Cruz, CA), β-galactosidase (1∶750, Abcam, Cambridge MA), Cytokeratin 8 (1∶100, University of Iowa Hybridoma Bank, Iowa City, IA), p63 (1∶50, Calbiochem, Gibbstown, NJ), androgen receptor (1∶50, Santa Cruz, CA), and CD45 (1∶50, BD Biosciences, San Jos, CA). Secondary anti-mouse or anti-rabbit Alexa Fluor 594 (red), Alexa Fluor 546 (orange-red) or Alexa Fluor 488 (green) were used as indicated (1∶500, Invitrogen, Carlsbad, CA). TO-PRO-3 iodide was used for confocal nuclear counterstaining (Invitrogen, Eugene, OR). DAPI mounting media was used for widefield immunofluorescence staining (Vector Laboratories, Burlngame, CA). Widefield images were taken on a Nikon epifluorescence microscope and a Leica DM IRB inverted microscope. Z-series slices were taken on a Zeiss LSM510 META inverted confocal microscope.

### Statistical Analysis

Oneway ANOVA was performed to test differences between the experimental and control groups, followed by Dunnett's post-hoc tests when the variances were equal. When variances were unequal, oneway Welch ANOVA allowing for unequal variances was used to test differences between the experimental groups and the intact group, followed by a two-sample t tests with unequal variances.
